# Multimodal Treatment With Orbital Organ Preservation in Adult Patients With Locally Advanced Small-Round-Cell Malignancy of the Nasal Cavity and Paranasal Sinus

**DOI:** 10.3389/fonc.2021.650385

**Published:** 2021-04-01

**Authors:** Nanxiang Chen, Hu Yuan, Wenjun Fan, Lin Ma, Kun Liu, Lei Chen, Shiming Yang, Xinxin Zhang

**Affiliations:** ^1^ ColIege of Otolaryngology Head and Neck Surgery, Chinese PLA General Hospital, National Clinical Research Center for Otolaryngologic Diseases, Key Lab of Hearing Science, Ministry of Education, Beijing Key Lab of Hearing Impairment for Prevention and Treatment, Beijing, China; ^2^ Affiliated Foshan Maternity & Child Healthcare Hospital, Southern Medical University, Foshan, China; ^3^ Department of Radiation Oncology, Nanfang Hospital, Southern Medical University, Guangzhou, China; ^4^ Department of Oncology, Armed Police Corps Hospital of Henan Province, Zhengzhou, China; ^5^ Department of Radiation Oncology, Chinese People’s Liberation Army General Hospital, Beijing, China

**Keywords:** multimodal treatment, rhabdomyosarcoma, olfactory neuroblastoma, neuroendocrine carcinoma, helical tomotherapy, small-round-cell, orbital preservation

## Abstract

**Background:**

To investigate the efficacy of induction chemotherapy followed by concurrent chemotherapy and helical tomotherapy in adult patients with locally advanced small-round-cell malignancy of the nasal cavity and paranasal sinus in regard to orbital organ preservation and quality of life.

**Methods:**

The clinical data of 49 patients with orbital involvement of locally advanced small-round-cell malignancy of the nasal cavity and paranasal sinus who received multimodal treatment for orbital organ preservation between December 2009 and January 2019 were retrospectively analyzed. Treatment efficacy and side effects were assessed. The study included three different pathological types. All patients were treated with induction chemotherapy followed by concurrent chemoradiotherapy. Helical tomotherapy was applied as radiotherapy. Adverse reactions to the chemotherapy were assessed according to Common Terminology Criteria for Adverse Events, Version 3. The overall survival (OS) rate, progression-free survival (PFS) rate, and orbital preservation rate were calculated using the Kaplan-Meier method.

**Results:**

After multimodal treatment, the 3- and 5-year OS rates of the 49 patients were 63.8% and 54.5%, respectively, and the 3- and 5-year total PFS rates were 66.8% and 63.1%, respectively.

**Conclusions:**

Multimodal treatment can preserve the orbital organs of adult patients with small-round-cell malignancy of the nasal cavity and paranasal sinus, achieve relatively ideal organ protection and survival rates, and improve quality of life, thus providing a new treatment option for these patients.

## Introduction

Among malignancies of the nasal cavity and paranasal sinuses, apart from the most common, squamous cell carcinoma, other types of malignancies are complex in pathological type and overlap in histological morphology. The biological behaviors of and clinical treatment strategies for these types are significantly different from those of nasal cavity and paranasal sinus squamous cell carcinoma. A considerable proportion of these lesions are small-round-cell malignancies or small-round-blue-cell malignancies based on cell morphology. Small-round-cell malignancies of the nasal cavity and paranasal sinuses include rhabdomyosarcoma (RMS), small-cell neuroendocrine carcinoma (NEC), olfactory neuroblastoma (ONB), mucosal malignant melanoma, Ewing’s sarcoma/peripheral primitive neuroectodermal tumor, lymphoid and hematopoietic malignancies, nasal undifferentiated carcinoma, and NUT carcinoma. Due to the low incidence and very small number of clinical cases, clarification of the pathological diagnosis of such malignancies and selection of the optimal clinical treatment are considerable challenges for pathologists and clinicians.

At most patients’ first visit, most lesions have invaded the orbit, intraocular muscles, and even intracranial structures. Although complete surgical removal of tumor tissue is the preferred radical approach (1), completely removing the tumor or obtaining a safe surgical margin is difficult because the anatomical structure of the nasal cavity and paranasal sinuses is sophisticated, and many important tissues and organs are clustered in this area (2). For stage T4 small-round-cell malignancies of the nasal cavity and paranasal sinuses that have invaded the eyes, to achieve complete resection of the malignancy, eyeball removal or even enlarged craniofacial resection is required, which seriously reduces patients’ life quality.

For patients with advanced small-round-cell malignancies of the nasal cavity and paranasal sinuses, maximally preserving eye function without affecting overall survival (OS) is a clinical problem urgently requiring resolution. Due to the low incidence of small-round-cell malignancies of the nasal cavity and paranasal sinuses, large-scale prospective randomized controlled multicenter clinical studies on the optimal treatment for the disease are currently lacking. Retrospective clinical studies have shown that patients receiving comprehensive treatment have a significantly better prognosis than patients receiving single-modality treatment (3). The National Comprehensive Cancer Network guidelines for sarcoma provide some treatment options for patients with stage IV cancer who are eligible for local treatment, such as stereotactic radiotherapy combined with chemotherapy, although the optimal treatment method requires further clinical research.

This study focused on three types of small-round-cell malignancies of the nasal cavity and paranasal sinuses: RMS, ONB, and small-cell NEC. The VID protocol, namely, vincristine (V), ifosfamide (I), and doxorubicin (D) chemotherapy, was combined with helical tomotherapy (HT) as a comprehensive treatment to protect the eyes in stage T4 adult patients with small-round-cell malignancies of the nasal cavity and paranasal sinuses invading the eyes. The 3-year OS rate, progression-free survival (PFS) rate, orbital preservation rate (OPR), and visual function preservation rate (FPR) and treatment-related side effects of all patients were retrospectively analyzed, and a stratified analysis according to pathological type was performed.

## Materials and Methods

### Ethical Approval

The study was conducted in accordance with the Declaration of Helsinki and was approved by the Ethics Committee of the PLA General Hospital. Individual informed consent was waived owing to the retrospective design.

### Study Design

#### Patient Information

A total of 49 patients with small-round-cell malignancies of the nasal cavity and paranasal sinuses who received full treatment in the Department of Otolaryngology Head and Neck Surgery of the PLA General Hospital from December 2009 to January 2019 were retrospectively analyzed in the study (patient information is listed in **Table 1**). All patients were above the age of 16. Seventeen patients underwent surgery under video endoscopy, all patients underwent partial resection, and residual malignancies were identified in the orbit apex, behind the eyeball, or in the cranial cavity (five patients experienced relapse rapidly within 1 month after surgery, and the sizes of the recurrent malignancies were larger than those of the primary malignancies at the time of admission). All three pathological types of malignancies were staged according to the eighth edition of the AJCC system. All patients were at stage T4, and the malignancies mainly invaded the orbit, intraocular muscles, skull base/dura mater, or intracranial structures. All patients expressed a strong desire to preserve their eyes and refused enucleation surgery. Prior to treatment, all patients were systemically assessed, including electrocardiogram, enhanced magnetic resonance imaging (MRI) of the nasal cavity and paranasal sinuses, nasal endoscopy, chest computed tomography (CT), ultrasound of cervical lymph nodes, abdominal ultrasound, and whole-body emission CT bone scans. Some patients underwent whole-body positron emission tomography–CT scans. Complete blood counts and biochemical profiles (liver and kidney function) before treatment were normal in all patients.

**Table 1:** Characteristics of the 49 evaluable patients.

Characteristics	No. of patients
Age, year, mean (range)	34.7 (16-65)
Sex, male:female	32:17
Follow-up, months, mean (range)	41.9 (7-107)
Loss to follow-up	2
Symptoms at diagnosis	
Nasal obstruction	35
Diplopia	31
Impaired vision/blindness	9/3
Headache	10
Epiphora	22
Proptosis	28
Epistaxis	29
T classification
T4a	38
T4b	11
N classification
N0	34
N1	15

#### Treatment Programs

Patients underwent multidisciplinary consultations with head and neck surgeons, oncologists, imaging doctors, and radiation therapists before treatment. The treatment plan was determined after comprehensive consideration of the patient’s sex, age, and general condition, tumor location, tumor size, the extent of involvement, regional lymph node metastasis. At the same time, all patients consulted with an ophthalmologist, and the patients’ eye function was evaluated through ophthalmological examination, enhanced MRI of the nasal cavity and paranasal sinuses, and vision and visual field examinations. All patients signed a chemotherapy consent form before treatment and were informed about and agreed to the treatment.

#### Chemotherapy

All patients received two or three cycles of induction chemotherapy in accordance with the VID protocol (i.e., V at 1 mg/m^2^ on day 1, I at 2.5 g/m^2^ on days 1-3, and D at 25 mg/m^2^ on days 1-2, with 21 days/cycle). The efficacy of induction chemotherapy was evaluated by enhanced MRI of the nasal cavity and paranasal sinuses. If a partial response (PR) or complete response (CR) was achieved, concurrent chemoradiotherapy was administered. During concurrent chemoradiotherapy, the VD protocol was used for chemotherapy: V at 1 mg/m^2^ on day 1 and D at 25 mg/m^2^ on days 1-2, with 21 days/cycle, for a total of three cycles. If a patient developed severe bone marrow suppression (≥ grade 3) during treatment, the chemotherapy doses were adjusted according to the lowest white blood cell (WBC) count after chemotherapy (**Table 2**).

**Table 2:** Chemotherapy dose adjustment.

Grade	Absolute neutrophil count	White blood cell count	Dose adjustment
1	>1.5	>3.0	Initial dose: D_x_ mg/m^2^
2	1.0-1.5	2.0-3.0	80%×D_x_ mg/m^2^
3	<1.0	1.0-2.0	70%×D_x_ mg/m^2^
4	<1.0	<1.0	50%-60%×D_x_ mg/m^2^

#### Radiotherapy

Radiotherapy was performed with HT (Hi-Art Tomotherapy; Accuray Inc., Sunnyvale, CA) for all patients. Patients underwent plain and enhanced CT scanning with 3-mm slice thickness and a thermoplastic mask for immobilization at first. CT images were then transmitted to a Pinnacle 3.8.0 treatment workstation (Philips Medical Systems, Fitchburg, WI, USA) and fused for target delineation. Gross target volumes of the primary tumor (GTVnx) and metastatic lymph node (GTVnd) were defined by the grossly visible tumor and metastatic lymphadenopathy on enhanced CT or MRI images. The planning GTVnx (pGTVnx) and planning GTVnd (pGTVnd) were obtained by expanding the corresponding GTVnx or GTVnd by 3 mm, which was limited by the brainstem, spinal cord, lenses, eyeballs, and optic nerve. The clinical target volume (CTV) included the nasal cavity, ethmoid sinus, frontal sinus, or maxillary sinus depending on the extent of tumor invasion and neck lymphatic drainage areas depending on the location of metastatic lymphadenopathy. Each CTV was automatically expanded to generate the corresponding planning target volume (PTV) with an isotropic 3-mm margin and at least 3 mm from the skin surface. Organs at risk (OARs) including the brainstem, spinal cord, bilateral lenses, eyeballs, optic nerve, inner ear, temporomandibular joint, and parotid glands as well as the oral cavity were also delineated. The margins of the CTV and PTV adjacent to critical OARs were modified accordingly.

CT images with contoured structures were transferred to an HT treatment planning workstation (Hi-Art Tomotherapy 2.2.4.1) for optimization. The total prescribed doses within the pGTVnx and pGTVnd were 66 to 70 Gy, while the dose within the PTV was 60 Gy, which were administered in 30-33 fractions. No more than 5% of the PTV volume received > 110% of the prescribed dose. The dose-volume planning constraints for OARs were as follows: (1) brainstem Dmax (maximum dose) < 54 Gy; (2) spinal cord Dmax < 45 Gy; (3) lens Dmax ≤ 8 Gy; (4) eye Dmax ≤ 50 Gy, Dmean (mean dose) < 35 Gy; (5) optic nerve Dmax ≤ 60 Gy; (7) temporomandibular joint Dmax ≤ 60 Gy; (8) parotid gland Dmean < 28 Gy; and (9) oral cavity V40 (the target volume receiving 40 Gy) < 30%. Radiation doses to orbit organs were kept as low as possible while ensuring that the target volume dose was met (**Table 3**).

**Table 3:** Dose-volume parameters of organs at risk (OARs) (mean ± SD).

OARs		D_max_(Gy)	D_min_(Gy)	D_mean_(Gy)	Volume(cm^3^)
Lens	Left	7.01 ± 1.19	5.03 ± 0.81	5.73 ± 0.88	0.20 ± 0.06
	Right	6.95 ± 1.28	4.96 ± 0.88	5.66 ± 0.99	0.21 ± 0.08
Eye	Left	52.78 ± 7.39	4.97 ± 0.91	21.75 ± 4.81	9.23 ± 1.66
	Right	52.08 ± 8.77	4.92 ± 1.10	21.31 ± 5.44	9.13 ± 1.83
Optic nerve	Left	66.44 ± 5.76	43.43 ± 9.51	58.78 ± 7.30	0.65 ± 0.27
	Right	65.04 ± 7.09	42.38 ± 9.46	57.40 ± 7.81	0.67 ± 0.30

Before each fraction of HT therapy, patients underwent megavoltage CT (MVCT) imaging to verify the patient setup. HT was delivered once daily to achieve five fractions per week and a total 30-33 fractions for 6-7 weeks.

#### Management of Major Adverse Events

Nasal endoscopy was performed before the first radiotherapy session and at the midterm assessment. Nasal irrigation, compound fish liver oil nasal drops, and antibiotic cream were used to prevent and treat grade 2 and 3 nasal mucosal membrane adhesion. If nasal mucosal membrane adhesion was identified, the adhesion region was separated in a timely manner. Radiation-induced oropharyngeal mucositis of grade 3 or higher was treated using a unique method employed in our department, that is, quinolone antibiotics + compound *Sophora flavescens* injection (4). All patients were closely monitored for complete blood counts, liver and kidney function, ions, albumin, and other conditions and were treated symptomatically.

### Statistical Analysis

Patient data were analyzed statistically using SPSS 25.0. OS and PFS curves and tumor local control survival curves were plotted with the Kaplan-Meier method.

### Evaluation of Efficacy and Adverse Responses

Efficacy was evaluated by a multidisciplinary collaboration group according to enhanced MRI of the nasal cavity and paranasal sinuses and the Response Evaluation Criteria in Solid Tumors (RECIST). Adverse responses to radiochemotherapy were assessed according to the third edition of the Common Terminology Criteria for Adverse Events (CTCAE). The OPR was defined as the proportion of patients who did not undergo enucleation surgery among patients who had survived for more than 3/5 years. The FPR was defined as the proportion of patients whose visual function did not decrease further from before treatment among patients who survived for more than 3/5 years.

### Follow-up

Follow-up information was collected from outpatient visits or telephone follow-ups. The first follow-up time was 1 month after the end of radiotherapy, and then the patients were followed up every 3 months within the first year, every 4 months within the second and third years, every 6 months within the fourth and fifth years, and once a year after 5 years. The last follow-up time was October 2019, and the total follow-up time was 7-107 months (mean 41.9 months, median 31 months). Two patients were lost to follow-up (after follow-ups of 33 and 45 months, respectively; each patient was free of disease at that time). The survival time of the patients was from the beginning of treatment to the last follow-up time or death.

## Results

### Efficacy Evaluation

**Figure 1:**
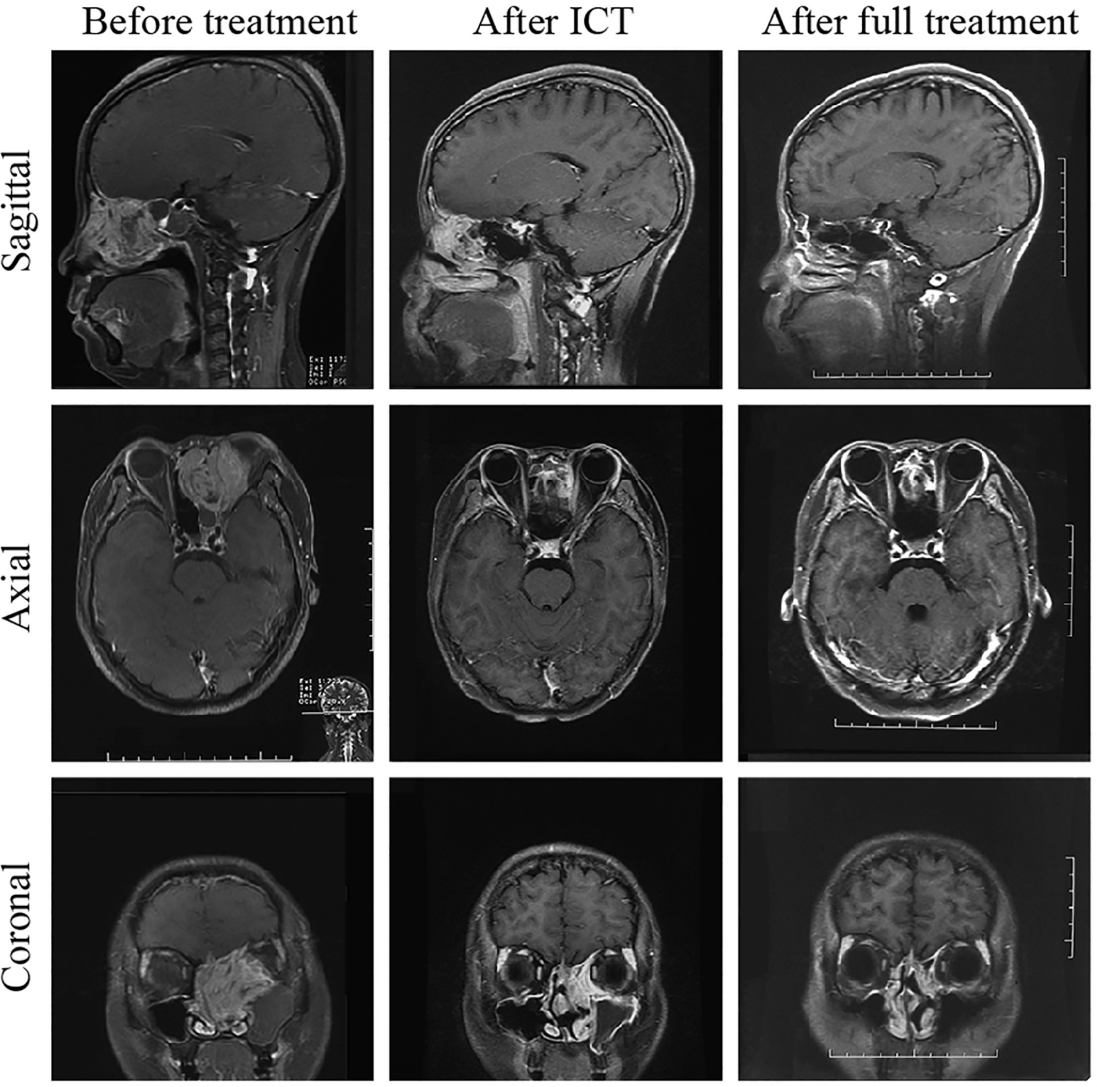
Comparison of MRI before and after treatment of one patient with rhabdomyosarcoma. The lesion size was significantly reduced after ICT; 1 month after treatment, the tumor had completely subsided. The patient was followed up for 52 months and remained free of disease.

**Figure 2:**
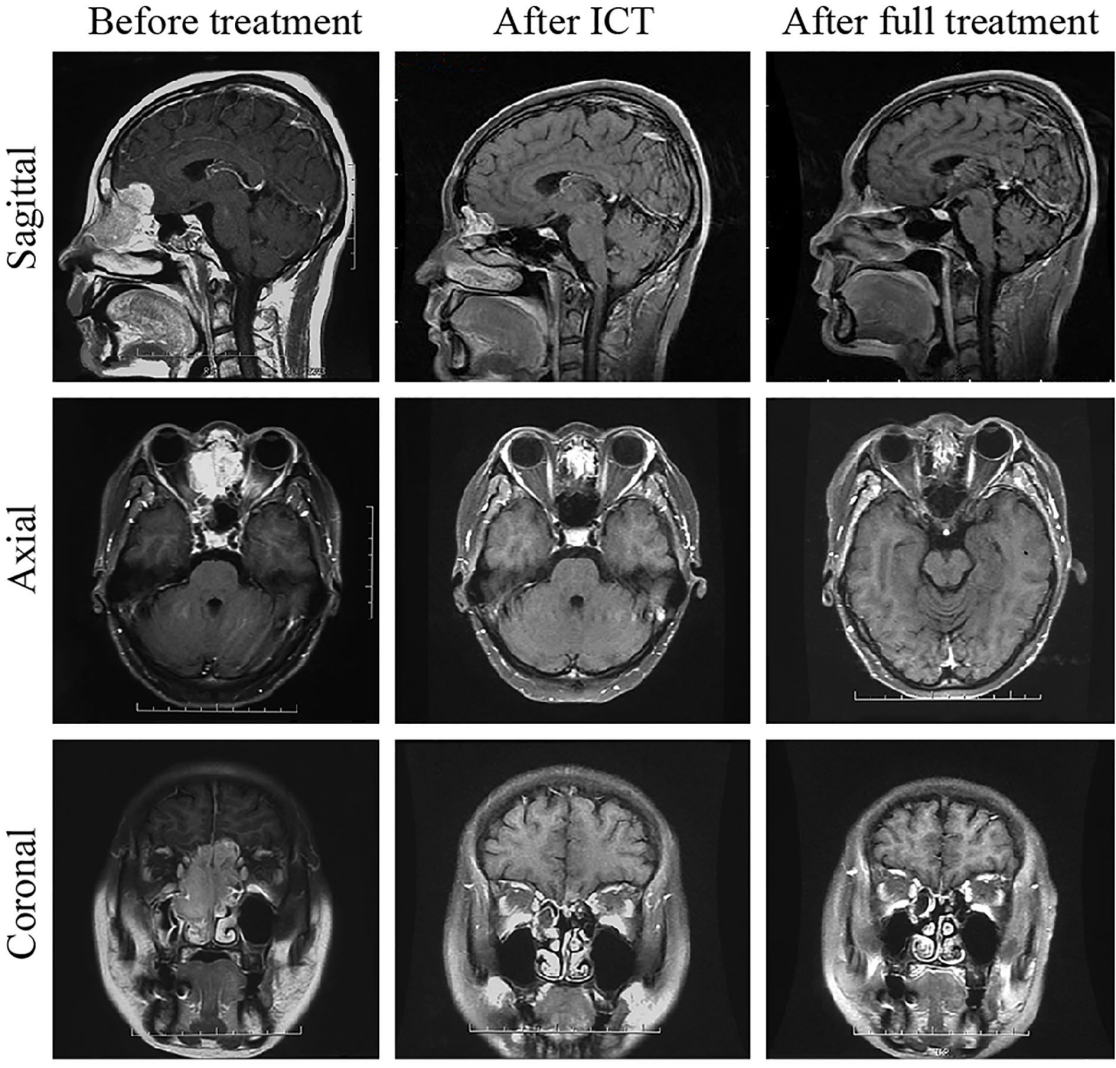
Comparison of MRI results before and after treatment of one patient with neuroendocrine carcinoma. One month after the treatment, the tumor had completely subsided. The patient was followed up for 54 months and remained free of disease.

Forty-five patients (45/49, 91.8%) completed the entire treatment cycle, and four patients (4/49, 8.2%) completed only two chemotherapy sessions during radiotherapy. A total of 13 patients developed grade 3 or higher bone marrow suppression/radiation-induced oral mucositis during radiotherapy, which resulted in an interrupted radiotherapy process with an average interruption time of 7.23 days (range: 2-13 days). During treatment, enhanced MRI of the nasal cavity and paranasal sinuses was used to evaluate changes in the lesions. Tumors significantly shrank after induction chemotherapy such that the malignancies recessed from the orbital apex, retrobulbar tissue, and craniocerebral tissue (**Figure 1**). Four patients (8.2%) achieved a CR, and 42 patients (85.7%) achieved a PR (smaller than the primary tumor by > 80%) and underwent concurrent chemoradiotherapy as scheduled. Three patients (6.1%) had a tumor size reduction greater than 50% but less than 80%, and the tumors did not detach from the orbital apex, retrobulbar tissue, and craniocerebral tissue. These patients received one more cycle of induction chemotherapy. After achieving a PR, these patients underwent concurrent chemoradiotherapy. The patients were examined 1 month after treatment was completed, and the giant tumors of the nasal cavity and paranasal sinuses of all 49 patients were found to reach a CR, yielding a treatment efficacy rate of 100%. **Figures 1** and **2** compare the MRI results before and after treatment of one patient with RMS and one patient with NEC, respectively.

### OS, PFS, the OPR, and the FPR

**Figure 3:**
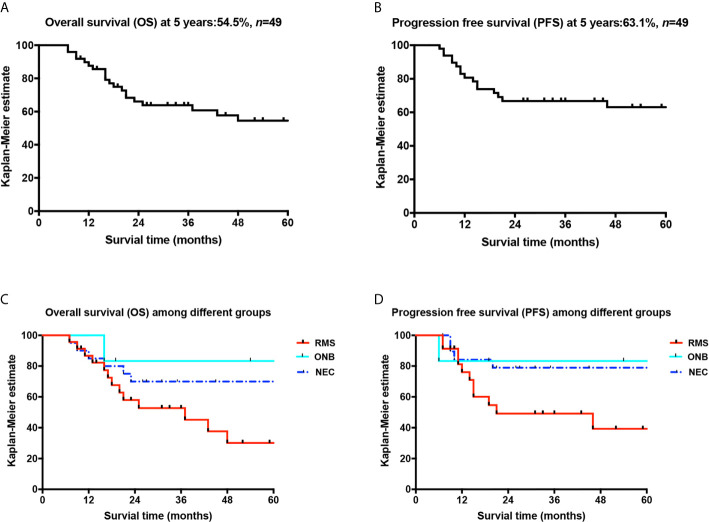
Overall survival curves of patients and comparison curves of different pathological types. **(A)** Overall survival curve; **(B)** progression-free survival curve; **(C)** comparison of the OS curves of the three pathological types; **(D)** comparison of the PFS curves of the three pathological types.

The 3- and 5-year OS rates of the 49 patients were 63.8% and 54.5%, respectively, and the 3- and 5-year total PFS rates were 66.8% and 63.1%, respectively. The 3- and 5-year OS rates of the 23 patients with RMS of the nasal cavity and paranasal sinuses were 52.7% and 30.1%, respectively, their 3- and 5-year PFS rates were 49.2% and 39.3%, respectively, and their 3-year OPR was 100%. The 5-year OS rate of the 20 patients with ONB was 70%, their 5-year PFS rate was 79%, and their 3-year OPR and FPR were 100% and 85% (11/13), respectively (including one patient with blindness in the right eye 48 months after treatment, one patient with binocular blindness 44 months after treatment, one patient with a follow-up less than 3 years, and six patients who survived less than 3 years). The 5-year OS rate of the six patients with NEC of the nasal cavity and paranasal sinuses was 83.3%, their 5-year PFS rate was 83.3% (see **Figure 3**), and the longest follow-up was 107 months free of disease; one patient showed vision loss after treatment, one patient died of meningeal metastasis at 16 months of follow-up, and the rest of the patients survived without disease recurrence. The total 3-year OPR was 100% (including one patient with embryonal RMS who had local recurrence 20 months after treatment and died without surgery). No patient developed eyeball atrophy after treatment. The total 3-year FPR was 89.8% (one patient had blindness in the right eye 48 months after treatment, one patient had binocular blindness 44 months after treatment, two patients had vision loss after treatment, and one patient developed diplopia 4 months after radiotherapy; the conditions of the above patients did not progress). By the end of the follow-up, 20 patients had died. Fifteen of these deaths were tumor-related deaths: two patients died of local recurrence (one patient with RMS did not undergo surgical treatment after recurrence and died 1 month after recurrence; one patient had lymph node metastasis in the parotid gland and received surgery and chemotherapy), and 13 patients died of distant metastases (including five cases of brain metastasis, two cases of pancreas, liver, and abdominal lymph node metastasis, one case of prostate metastasis, three cases of lung metastasis, and two cases of bone metastasis). Among the patients with distant metastases, one patient with RMS who died of lung metastasis was found to have local recurrence on the left side at 14 months and underwent palliative surgery. Recurrence was found again at 21 months after treatment, and the patient underwent surgical treatment. Lung metastasis appeared at 34 months. This patient discontinued treatment and died at 37 months of follow-up. Of the other 12 patients who were found to have distant metastases, 10 patients received salvage chemotherapy, and two declined further treatment. One death was treatment-related, resulting from bone marrow suppression after treatment, with a survival time of 7 months. Four other patients died of unknown causes.

### Major Toxicities and Side Effects During Treatment

Adverse responses to radiotherapy and chemotherapy were evaluated using the third edition of the CTCAE. Adverse responses during treatment included vision-related adverse responses (**Table 4**) and non-vision-related adverse responses (**Table 5**). Among them, vision-related adverse responses below grade 2 mainly included xeroma (32.6%) and conjunctivitis (14.3%), while vision-related adverse responses above grade 3 included optic neuropathy (one case) and extraocular muscle paralysis (one case). Non-vision-related adverse responses of grades 2 and 3 mainly included nonhematological adverse responses and hematological adverse responses. The nonhematological adverse responses mainly included radiation-induced oropharyngeal mucositis (89.8%), radiation dermatitis (77.6%), weight loss (63.3%), and nasal obstruction (67.4%). Due to severe side effects during concurrent chemoradiotherapy, radiotherapy was interrupted in 13 patients. The main causes of nasal obstruction were crusting and adhesion of the mucous membrane and dryness of the nasal cavity. Nasal mucous membrane adhesion was significant in two patients, and timely separation of adhesion was performed; thus, ventilation function was not affected. Non-vision-related adverse responses during induction chemotherapy included grade 2 alopecia, grade 1-2 emesis, etc., with mild symptoms; more serious hematological adverse responses included grade 3-4 WBC count reductions, including grade 3 leukopenia in 23 cases (46.9%) and grade 4 leukopenia in two cases (4.1%). Throughout the full course of treatment, grade 3-4 hematological adverse responses mainly included bone marrow suppression, including grade 3 leukopenia in 32 cases (65.3%), grade 4 leukopenia in nine cases (18.4%), grade 3 neutropenia in 37 patients (75.5%), grade 4 neutropenia in six patients (12.2%), and grade 4 thrombocytopenia in three patients (6.1%). One patient died due to bone marrow suppression without timely treatment after radiochemotherapy.

**Table 4:** Incidence of vision-related adverse responses.

Adverse response	Number of cases (%)			
	Grade 1	Grade 2	Grade 3	Grade 4
Acute adverse responses				
Conjunctivitis	5 (10.2)	2 (4.1)	0	0
Keratitis	2 (4.1)	1 (2)	0	0
Epiphora	6 (12.2)	0	0	0
Delayed adverse responses				
Xeroma	13 (26.5)	3 (6.1)	0	0
Optic neuropathy	0	1 (2)	1 (2)	0
Extraocular muscle paralysis	0	0	1 (2)	0

**Table 5:** Incidence of non-vision-related adverse responses.

Adverse response	Number of cases (%)			
	Grade 1	Grade 2	Grade 3	Grade 4
Nonhematological adverse responses				
Oral mucositis	5 (10.2)	13 (26.5)	31(63.3)	0
Paranasal sinus disease	3 (6.1)	7 (14.2)	0	0
Radiation dermatitis	11 (22.4)	29 (59.2)	9 (18.4)	0
Nose obstruction	16 (32.7)	19 (38.8)	14 (28.6)	0
Tinnitus	13 (26.5)	3 (6.1)	0	0
Hearing impairment	7 (14.2)	5 (10.2)	2 (4.1)	0
Weight loss	8 (16.3)	18 (36.7)	7 (14.3)	0
Alopecia	11 (22.4)	38(77.5)	0	0
Emesis	39 (79.6)	8 (16.3)	2 (4.1)	0
Hematological adverse responses				
Leukopenia	0	8 (16.3)	32 (65.3)	9 (18.4)
Neutropenia	0	6 (12.2)	37 (75.5)	6 (12.2)
Febrile neutropenia	0	0	13 (26.5)	0
Thrombocytopenia	6 (12.2)	21 (42.9)	19 (38.8)	3 (6.1)

## Discussion

Locally advanced malignancies of the nasal cavity and paranasal sinuses can invade the orbits *via* the surrounding thin bone plates to cause eyeball protrusion, vision loss, eye movement disorders, and even blindness. Preserving the visual function of such patients and avoiding destructive surgery while ensuring survival constitute the goal and research direction that we have always pursued. Squamous cell carcinoma is the most common pathological type of malignancy of the nasal cavity and paranasal sinuses. We have published the results of a single-center study on the clinical treatment of patients with locally advanced squamous cell carcinoma of the nasal cavity and paranasal sinuses that had invaded the eyes and have achieved satisfactory results from eye-preserving treatment for patients with locally advanced squamous cell carcinoma while ensuring survival (5).

For the large category of small-round-cell malignancies of the nasal cavity and paranasal sinuses, the treatment methods and prognoses differ depending on the pathological type, and describing them uniformly is difficult. Preliminary results in this study showed that for adult patients with locally advanced small-round-cell malignancies, this treatment method resulted in a high response rate and good protection of OARs, such as the lens and optic nerves, and yielded a high OPR and FPR.

### Rhabdomyosarcoma

The largest-scale analyses of adult RMS of the nasal cavity and paranasal sinuses have been conducted by Stepan et al. (6) and Unsal et al. (7), who retrospectively analyzed the data of 186 cases of adult RMS of the nasal cavity and paranasal sinuses from 2004 to 2013 in the National Cancer Database of the United States, resulting in a 5-year OS rate of 28.4%. Unsal et al. (7) analyzed 286 patients of all ages with RMS of the nasal cavity and paranasal sinuses from 1973 to 2013 in the Surveillance, Epidemiology, and End Results (SEER) Database and reported an overall 5-year survival rate of 35.1% (including 172 adult patients; the 5-year OS rate ranged from 17.8% to 24.6% in different age groups). In their study, the 3- and 5-year OS rates of 23 adult patients with stage T4 (six patients in stage T4b) RMS of the nasal cavity and paranasal sinuses were 52.7% and 30.1%, respectively, and the OPR was 100% (**Table 6**). However, the follow-up times of the 23 patients were mostly less than 5 years; thus, the 5-year OS rate might be skewed. Under the premise of ensuring survival, this treatment method enables patients to have a higher quality of life and lowers the incidence of vision-related side effects.

**Table 6:** Comparison of OS and OPRs with different regimens for sinonasal malignancies.

Study	Histological type (Number of cases)	Treatment regimen (Number of cases)	OS	OPRs
Stepan et al. (6)	RMS (186 adults)	Chemo+RT (90) Surgery+Chemo+RT (47) Chemo Only (31) Other (14)	28.4% (5-year) 22.6% (5-year)*	–
Unsal et al. (7)	RMS (23 T4 adults)	Surgery alone (−) Surgery+ RT (−)	52.7% (3-year) 30.1% (5-year)	100%
Bartel et al. (8)	ONB (6 Kadish C)	IC+ Surgery+RT (4) Radical surgery (2)	88.9% (5-year)	66.6%
Su SY et al. (9)	ONB (12 stage T4)	IC+ RT/+Surgery/+CCRT	78% (5-year)	–
Rosenthal et al. (10)	NEC (18 T2-T4)	Surgery+ RT (8) IC+ Surgery or RT (8)	64.2% (5-year)	–
Mitchell et al. (11)	NEC (21 stage IV)	Surgery+ RT (5) Surgery (6) Chemoradiation (10)	56.8% (5-year)	–
The present study	RMS (23 T4) ONB (20 T4) NEC (6 T4)	IC+CCRT	30.1% (5-year) 70% (5-year) 83.3% (5-year)	100%

*With intracranial extension, CCRT, concurrent chemoradiotherapy; Chemo, chemotherapy; RT, radiotherapy; IC, induction chemotherapy

### Olfactory Neuroblastoma

For advanced patients with Kadish stage C or above or Dulguerov TNM stage T3 or above, comprehensive treatment can benefit patients significantly more than single-modality therapy. At present, except for Kadish stage A patients who can be treated with surgery alone, routine treatment strategies for patients at other stages usually include radical surgery + postoperative radiotherapy (12). However, research on the optimal comprehensive treatment plan has never stopped. Bartel et al. (8) conducted a retrospective study of nine patients with ONB of the nasal cavity and paranasal sinuses (six patients at Kadish stage C) and concluded that induction chemotherapy played an important role in reducing tumor size, obtaining safe resection margins, and minimizing complications. The MD Anderson Cancer Center treated 15 patients with ONB of the nasal cavity and paranasal sinuses (including 12 patients at stage T4) with induction chemotherapy followed by radical treatment (radical radiotherapy, surgery, and concurrent chemoradiotherapy) and achieved 5-year total DFS and total OS rates of 71% and 78%, respectively. The results suggest that ONB is sensitive to chemotherapy and that induction chemotherapy for locally advanced patients is an acceptable treatment (9). In this study, 20 patients with ONB (five at stage T4b) showed a 5-year OS rate of 70%, a 5-year PFS rate of 79%, and a FPR of 85%, indicating that eye function was effectively preserved on the basis of ensuring survival.

### Neuroendocrine Carcinoma

NEC, similar to ONB, is a malignancy of the nasal cavity and paranasal sinuses with neuroendocrine differentiation, accounting for approximately 5% of malignancies of the nasal cavity and paranasal sinuses. Rosenthal et al. (10) performed induction chemotherapy on eight of 18 patients with NEC and achieved a 5-year survival rate of 64.2%. They believed that NEC was sensitive to chemotherapy and prone to distant metastasis; therefore, induction chemotherapy + concurrent chemoradiotherapy or surgery + postoperative radiotherapy was the treatment of choice. Mitchell et al. (11) analyzed 28 patients with NEC of the nasal cavity and paranasal sinuses treated in the MD Anderson Cancer Center from 1990 to 2004, and their 5-year OS rate was 65%. Since NEC is a small-round-cell malignancy, these authors selected the VID protocol for chemotherapy of NEC, and the 5-year OS rate was 83.3%, with only one patient who experienced slight vision loss, demonstrating satisfactory efficacy.

Under the premise of ensuring survival, we obtained high OPRs and FPRs, which were related to the choice of treatment regimen. Because the maximum tolerated doses of involved organs (brain stem < 54 Gy, spine < 45 Gy, lens ≤ 8 Gy, eyeball < 35 Gy (mean), and optic nerves ≤ 60 Gy) are substantially lower than the dose for radical irradiation of the tumor target (66-70 Gy), radiotherapy cannot be directly used. In this study, the VID protocol was used for induction chemotherapy, and the tumors significantly shrank after induction chemotherapy, causing them to detach from the orbital apex and the retrobulbar region, which provided a greater safety margin around the target area. This strategy not only reduced radical radiotherapy-induced damage to the eyeball and optic nerves and protected the eyes but also significantly increased sensitivity to radiotherapy through induction chemotherapy. Second, for stage T4b patients with intracranial tumor invasion, induction chemotherapy can shrink the intracranial tumor, causing it to recede from the brain tissue and skull base (stage T4b NEC shown in **Figure 2**), which provides an opportunity for radical radiotherapy of the tumor and thus changes palliative treatment to radical treatment. More importantly, this study employed a treatment method of induction chemotherapy + concurrent chemoradiotherapy, which improved the local control survival rate and reduced the local recurrence rate, demonstrating an important role in ensuring survival and improving the OPR. At the same time, to minimize damage to important surrounding organs and structures by radiotherapy, HT technology was applied to all patients in this study to protect the eyes of the stage T4 patients. HT technology not only ensures that the target area receives a higher conformal radiotherapy dose but also sharply reduces the radiation dose to the normal tissue surrounding the target area (13, 14), thereby controlling the radiation dose to involved organs (optic nerves, lens, etc.) within a tolerable range while ensuring a full total radiotherapy dose and thus protecting the visual pathway and reducing the incidence of visual adverse responses, such as severe xeroma and blindness caused by radiotherapy. Compared with regular two-dimensional (2D) radiotherapy and conventional conformal radiotherapy, HT radiotherapy technology has obvious advantages (15, 16), which is one of the reasons for the high OPR in this group of patients (**Table 6**).

Radiotherapy plays an important in the treatment of cancers of the paranasal sinus and nasal cavity, especially for patients with unresectable lesions. With advances from conventional 2D radiotherapy to three-dimensional (3D) conformal radiotherapy and further to intensity-modulated radiation therapy (IMRT), improvements in clinical outcomes have paralleled the technological gains that have been achieved. The proportion of patients with cancers of the paranasal sinus and nasal cavity who survived for 5 years (regardless of the type of radiotherapy) increased from 28% in the 1960s to 51% in the 1990s (17). Meanwhile, the rates of grade 3 or greater visual toxicity apparently decreased from 53% in the 1960s to 16% in the 2000s (18). However, the survival benefits and the incidence of late toxicity for patients with cancers of the paranasal sinus and nasal cavity receiving IMRT are not satisfactory.

In the last two decades, more advanced radiotherapy techniques such as HT and charged particle therapy with protons, helium ions, carbon ions, or neon ions have been increasingly applied in the treatment of head and neck cancers. HT relies on inverse planning but uses a rotational gantry system rather than a fixed number of beam angles, as in traditional segmental multileaf collimator-based IMRT, for radiation delivery. Compared to IMRT, HT provides better conformity and dose homogeneity, which may achieve substantial dose reductions to OARs without compromising dose delivery to the tumor target (19, 20). However, studies of HT in cancers of the paranasal sinus and nasal cavity are scarce. Our center previously conducted a retrospective study to investigate the efficacy of multimodal treatment including HT in patients with locally advanced squamous cell carcinoma of the nasal cavity and paranasal sinus in regard to orbital organ preservation and quality of life, and the results showed a 3-year OS rate of 59.2%, a local control rate of 80.2%, and a rate of effective orbital preservation of 77.8% (5). In this study focusing on locally advanced small-round-cell malignancies of the nasal cavity and paranasal sinus, radiation doses to the lens, eyes, and optic nerves were slightly lower than those in our previous study, and only two of 49 patients developed grade 3 visual toxicity. Moreover, the 3- and 5-year OS rates were 63.8% and 54.5%, and the 3- and 5-year total PFS rates were 66.8% and 63.1%, respectively. Although the results of our study showed improved advantages of HT, prospective studies of HT versus IMRT in the treatment of cancers of the nasal cavity and paranasal sinus are still needed.

Charged particle therapy has a theoretical advantage of a rapid dose fall-off beyond the Bragg peak (sharp accumulation of the dose at a specific depth in tissue), which allows more conformal treatment with better targeted dose coverage of the tumor (21). This improvement further allows dose escalation to the tumor and apparent dose reductions to adjacent organs. Some studies have investigated charged particle therapy (especially proton therapy) for the treatment of cancers of the nasal cavity and paranasal sinus. Patel et al. (22) performed a systematic review and meta-analysis to compare the clinical outcomes of patients with cancers of the paranasal sinuses and nasal cavity (including all malignant histological types except for lymphomas) treated with charged particle therapy (including particle therapy with protons, helium ions, carbon ions, or neon ions) with those of individuals receiving photon therapy including 2D, 3D, or IMRT techniques. The use of photon therapy resulted in a 5-year OS rate of 48% and a DFS rate of 41%, while the use of charged particle therapy resulted in higher 5-year OS (72%) and DFS rates (80%) with significance differences (P < 0.003 for both). However, charged particle therapy yielded no improvements of toxicity to the eyes, ears, nasal membranes, and miscellaneous structures and showed even higher neurological toxicity than photon therapy. In a subgroup analysis of proton therapy versus IMRT, a benefit of proton beam therapy with respect to 5-year OS (66% vs 48%, P = 0.057) and DFS (72% vs 50%, P = 0.045) was observed. This remarkable result suggests that the theoretical advantage of proton therapy may be real for the treatment of cancers of the nasal cavity and paranasal sinuses. However, prospective studies comparing proton therapy and IMRT or proton therapy and HT are lacking. Our future studies will explore these issues.

## Data Availability Statement

The raw data supporting the conclusions of this article will be made available by the authors, without undue reservation.

## Ethics Statement

The study was approved by the ethics committee of the PLA General Hospital. The patients/participants provided their written informed consent to participate in this study. Written informed consent was obtained from the individual(s) for the publication of any potentially identifiable images or data included in this article.

## Author Contributions

NC, HY and WF contributed equally to this work. NC, LM, SY, and XZ contributed to the conception of this study. All authors participated in the design and the writing of the study. NC, HY, WF, KL, and XZ examined the archives and identified the cases included in the study. NC, HY, LC, and XZ performed clinical case collection for the study. All authors contributed to the article and approved the submitted version.

## Conflict of Interest

The authors declare that the research was conducted in the absence of any commercial or financial relationships that could be construed as a potential conflict of interest.

## References

[1] Mendenhall WM Mendenhall CM Werning JW Riggs CE Mendenhall NP. Adult head and neck soft tissue sarcomas. Head Neck (2005) 27:916–22. 10.1002/hed.20249 16136585

[2] de Bree R van der Waal I de Bree E Leemans CR. Management of adult soft tissue sarcomas of the head and neck. Oral Oncol (2010) 46:786–90. 10.1016/j.oraloncology.2010.09.001 20947413

[3] Gore MR. Treatment, outcomes, and demographics in sinonasal sarcoma: a systematic review of the literature. BMC Ear Nose Throat Disord (2018) 18:4. 10.1186/s12901-018-0052-5 29581706 PMC5861608

[4] Zhang XX Ma L Wang JL Wu WM Feng LC Huang DL. Management of oral mucositis in patients with head and neck cancer receiving chemoradiotherapy and/or molecular targeted therapy. Zhonghua Er Bi Yan Hou Tou Jing Wai Ke Za Zhi (2011) 46:505–8. 21924104

[5] Chen NX Chen L Wang JL Wang JY Yan F Ma L. A clinical study of multimodal treatment for orbital organ preservation in locally advanced squamous cell carcinoma of the nasal cavity and paranasal sinus. Jpn J Clin Oncol (2016) 46:727–34. 10.1093/jjco/hyw064 27207888

[6] Stepan K Konuthula N Khan M Parasher A Del Signore A Govindaraj S. Outcomes in adult sinonasal rhabdomyosarcoma. Otolaryngol Head Neck Surg (2017) 157:135–41. 10.1177/0194599817696287 28669309

[7] Unsal AA Chung SY Unsal AB Baredes S Eloy JA. A population-based analysis of survival for sinonasal rhabdomyosarcoma. Otolaryngol Head Neck Surg (2017) 157:142–9. 10.1177/0194599817696292 28397540

[8] Bartel R Gonzalez-Compta X Cisa E Cruellas F Torres A Rovira A. Importance of neoadjuvant chemotherapy in olfactory neuroblastoma treatment: series report and literature review. Acta Otorrinolaringol Esp (2018) 69:208–13. 10.1016/j.otoeng.2017.07.003 29061289

[9] Su SY Bell D Ferrarotto R Phan J Roberts D Kupferman ME. Outcomes for olfactory neuroblastoma treated with induction chemotherapy. Head Neck (2017) 39:1671–9. 10.1002/hed.24822 28561956

[10] Rosenthal DI Barker JL Jr. El-Naggar AK Glisson BS Kies MS Diaz EM Jr. Sinonasal malignancies with neuroendocrine differentiation: patterns of failure according to histologic phenotype. Cancer (2004) 101:2567–73. 10.1002/cncr.20693 15517582

[11] Mitchell EH Diaz A Yilmaz T Roberts D Levine N DeMonte F. Multimodality treatment for sinonasal neuroendocrine carcinoma. Head Neck (2012) 34:1372–6. 10.1002/hed.21940 22052583

[12] Ow TJ Hanna EY Roberts DB Levine NB El-Naggar AK Rosenthal DI. Optimization of long-term outcomes for patients with esthesioneuroblastoma. Head Neck (2014) 36:524–30. 10.1002/hed.23327 23780581

[13] Bauman G Yartsev S Rodrigues G Lewis C Venkatesan VM Yu E. A prospective evaluation of helical tomotherapy. Int J Radiat Oncol Biol Phys (2007) 68:632–41. 10.1016/j.ijrobp.2006.11.052 17321068

[14] Adams EJ Nutting CM Convery DJ Cosgrove VP Henk JM Dearnaley DP. Potential role of intensity-modulated radiotherapy in the treatment of tumors of the maxillary sinus. Int J Radiat Oncol Biol Phys (2001) 51:579–88. 10.1016/S0360-3016(01)01655-8 11597796

[15] Lai SZ Li WF Chen L Luo W Chen YY Liu LZ. How does intensity-modulated radiotherapy versus conventional two-dimensional radiotherapy influence the treatment results in nasopharyngeal carcinoma patients? Int J Radiat Oncol Biol Phys (2011) 80:661–8. 10.1016/j.ijrobp.2010.03.024 20643517

[16] Peng G Wang T Yang KY Zhang S Zhang T Li Q. A prospective, randomized study comparing outcomes and toxicities of intensity-modulated radiotherapy vs. conventional two-dimensional radiotherapy for the treatment of nasopharyngeal carcinoma. Radiother Oncol (2012) 104:286–93. 10.1016/j.radonc.2012.08.013 22995588

[17] Dulguerov P Jacobsen MS Allal AS Lehmann W Calcaterra T. Nasal and paranasal sinus carcinoma: are we making progress? A series of 220 patients and a systematic review. Cancer (2001) 92:3012–29. 10.1002/1097-0142(20011215)92:12<3012::AID-CNCR10131>3.0.CO;2-E 11753979

[18] Chen AM Daly ME Bucci MK Xia P Akazawa C Quivey JM. Carcinomas of the paranasal sinuses and nasal cavity treated with radiotherapy at a single institution over five decades: are we making improvement? Int J Radiat Oncol Biol Phys (2007) 69:141–7. 10.1016/j.ijrobp.2007.02.031 17459609

[19] Fiorino C Dell’Oca I Pierelli A Broggi S De Martin E Di Muzio N. Significant improvement in normal tissue sparing and target coverage for head and neck cancer by means of helical tomotherapy. Radiother Oncol (2006) 78:276–82. 10.1016/j.radonc.2006.02.009 16546279

[20] Chen AM Sreeraman R Mathai M Vijayakumar S Purdy JA. Potential of helical tomotherapy to reduce dose to the ocular structures for patients treated for unresectable sinonasal cancer. Am J Clin Oncol (2010) 33:595–8. 10.1097/COC.0b013e3181c44535 20142725

[21] van de Water TA Bijl HP Schilstra C Pijls-Johannesma M Langendijk JA. The potential benefit of radiotherapy with protons in head and neck cancer with respect to normal tissue sparing: a systematic review of literature. Oncologist (2011) 16:366–77. 10.1634/theoncologist.2010-0171 PMC3228110 21349950

[22] Patel SH Wang Z Wong WW Murad MH Buckey CR Mohammed K. Charged particle therapy versus photon therapy for paranasal sinus and nasal cavity malignant diseases: a systematic review and meta-analysis. Lancet Oncol (2014) 15:1027–38. 10.1016/S1470-2045(14)70268-2 24980873

